# Effect of ketamine combined with magnesium sulfate in neuropathic pain patients (KETAPAIN): study protocol for a randomized controlled trial

**DOI:** 10.1186/s13063-017-2254-3

**Published:** 2017-11-03

**Authors:** Noémie Delage, Véronique Morel, Pascale Picard, Fabienne Marcaillou, Bruno Pereira, Gisèle Pickering

**Affiliations:** 1 Centre d’Evaluation et de Traitement de la Douleur, CHU de Clermont-Ferrand, F-63003 Clermont-Ferrand, France; 2 Centre de Pharmacologie Clinique, Bâtiment 3C, CIC Inserm 1405, CHU Clermont-Ferrand, BP 69, F-63003 Clermont-Ferrand, Cedex 1 France; 3 CHU de Clermont-Ferrand, Délégation Recherche Clinique & Innovation - Villa annexe IFSI, 58 Rue Montalembert, F-63003 Clermont-Ferrand, Cedex France; 4 Inserm, U1107 Neuro-Dol, Pharmacologie Fondamentale et Clinique de la Douleur, Laboratoire de Pharmacologie, Faculté de Médecine, Université Clermont Auvergne, F-63000 Clermont-Ferrand, France

**Keywords:** Ketamine, Neuropathic pain, Magnesium sulfate, *N*-methyl-d-aspartate receptor, Placebo

## Abstract

**Background:**

Neuropathic pain is difficult to treat, and the efficacy of recommended drugs remains limited. *N*-methyl-d-aspartate receptors are implicated, and antagonists are a pharmacological option. Ketamine is widely used in French pain clinics, but without consensus or recommendations. Furthermore, the association of ketamine with magnesium has been poorly studied. The aim of the present study is to evaluate the benefit of ketamine with or without magnesium in refractory neuropathic pain.

**Methods/design:**

A randomized, double-blind, crossover, placebo-controlled study will be performed in Clermont-Ferrand University Hospital, Clermont-Ferrand, France. The aim is to evaluate the effect of ketamine with or without magnesium in 22 patients with neuropathic pain. Intravenous ketamine/placebo, ketamine/magnesium sulfate, or placebo/placebo will be administered consecutively to each patient, in random order, once at 5-week intervals. The primary endpoint is the AUC of pain intensity assessed on a 0–10 Numeric Pain Rating Scale for a 5-week period. Data analysis will be performed on an intention-to-treat basis, and all statistical tests (except primary analysis) will be performed with an α risk of 5% (two-sided).

**Discussion:**

Considering the poor efficacy of the drugs available for neuropathic pain, ketamine with or without magnesium sulfate may be a valuable therapeutic option that needs to be standardized.

**Trial registration:**

EudraCT number–2015-000142-29. Registered on April 9, 2015; version 1.4

**Electronic supplementary material:**

The online version of this article (doi:10.1186/s13063-017-2254-3) contains supplementary material, which is available to authorized users.

## Background

Neuropathic pain (NP), whatever the etiology or topography, presents with a number of clinical characteristics: sensory deficit, hypoesthesia, electric shock-like pain, allodynia, or hyperalgesia [1]. *N*-methyl-d-aspartate receptors (NMDARs) are involved in the development of NP and may provide an avenue to a pharmacological tool for treatment. Ketamine, an NMDAR antagonist, has been used for a number of years for the treatment of NP. In animals, it decreases NP symptoms [2–10]. In humans, ketamine at infra-anesthetic doses has shown some efficacy in postoperative pain [11], refractory pain [12], phantom limb pain [13], postherpetic neuralgia [14], and complex regional pain syndrome (CRPS) [15–17]. Most clinical studies showing efficacy were retrospective [18–21], open prospective [22, 23], or clinical reports [24, 25]. However, a few randomized, placebo-controlled, double-blind studies have also been published [15–17]. Ketamine appears to be well tolerated at low doses, with little psychodysleptic effect [16, 19, 20, 22, 23].

Another NMDAR modulator, magnesium sulfate, is a physiological blocker of NMDAR and has also been shown to be effective in the treatment of NP. In animal NP models, it decreased cold allodynia and thermal and mechanical hyperalgesia [26–29] and increased the analgesic effect of morphine [27, 30]. In humans, it is well tolerated and has shown efficacy against NP in patients with cancer [31], headache [32], and postoperative pain [33], as well as in the frequency of pain paroxysms and in the emotional component [34].

Concerning the ketamine-magnesium sulfate interaction, a preclinical study showed a synergistic effect, blocking the hyperthermia caused by systemic administration of morphine [35]. A recent randomized, prospective, double-blind study [36] of patients undergoing scoliosis surgery showed that magnesium sulfate combined with ketamine had a greater beneficial effect on postoperative pain and morphine consumption than ketamine alone. Furthermore, patients receiving ketamine combined with magnesium sulfate reported better sleep. Magnesium sulfate and ketamine act on different sites of the NMDAR channel, and this could explain their synergistic role. This combination is hypothesized to reduce ketamine dosage and diminish its adverse events, although another randomized, double-blind, controlled trial [37] showed no significant difference in the occurrence of adverse events among patients receiving ketamine either alone or combined with magnesium sulfate. Given the lack of randomized clinical trials of ketamine or the combination of ketamine and magnesium, the objective of the present randomized, controlled, double-blind trial is to assess the efficacy of intravenous ketamine with or without magnesium in patients with NP.

## Methods/design

**Fig. 1:**
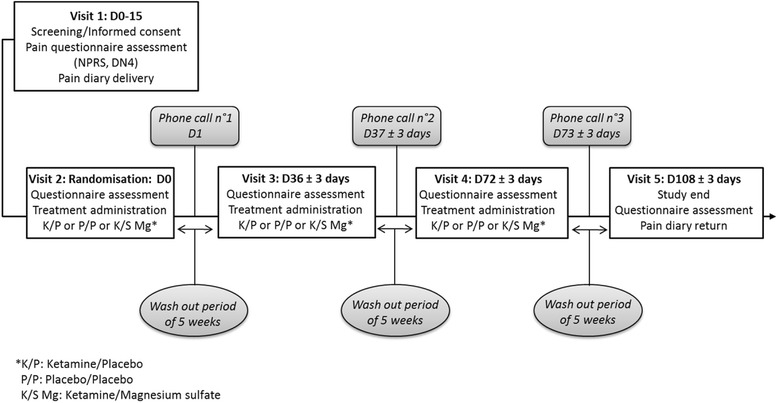
Study design. *DN4* Diagnosing Neuropathic Pain 4 Questions, *NPRS* Numeric Pain Rating Scale

This is a randomized, placebo-controlled, double-blind, crossover, single-center clinical study with intravenous ketamine/placebo, ketamine/magnesium sulfate, and placebo/placebo injections given to each patient. This clinical trial is reported according to the Standard Protocol Items: Recommendations for Interventional Trials (SPIRIT) guidelines (*see* Additional file 1) and performed in Clermont-Ferrand University Hospital, Clermont-Ferrand, France. Patients meeting the inclusion criteria will sign a consent form after receiving oral and written information from a physician investigator involved in this project. During this consultation, they will fill out a Numeric Pain Rating Scale (NPRS), as well as the Diagnosing Neuropathic Pain 4 Questions [38] in order to detect NP, and they will be asked to keep a daily pain diary (mean daily pain and maximum pain on NPRS, and concomitant analgesic treatment) for 14 days before the first course of treatment. After inclusion, patients will be randomly assigned to ketamine-placebo, ketamine-magnesium sulfate, or placebo-placebo for 1 month, in random order, with a washout period of 5 weeks between every treatment (Fig. 1). At each injection, the patients will undergo a number of tests for assessment of (1) pain (NPRS, Brief Pain Inventory [BPI] [39], McGill Pain Questionnaire [40], and Patient Global Impression of Change [PGIC] [41]), (2) NP (Neuropathic Pain Symptom Inventory [NPSI] [42]), (3) anxiety and depression (Hospital Anxiety and Depression Scale [HADS] [43], (4) quality of life (36-item Short Form Health Survey [SF-36] [44]), and (5) quality of sleep (Pittsburgh Sleep Quality Index [PSQI] [45]). All questionnaires will be administered before each ketamine injection in order not to influence the results because of the possible ketamine adverse events. To check for any adverse events, subjects will be called by phone on the day following each injection.

### Treatment

#### Ketamine

Ketamine will be administered in ampoules of 50 mg/5 ml intravenously with an electric syringe at the dose of 0.5 mg/kg diluted in 45 ml of physiological saline (0.9% NaCl) according to the usual procedures of the pain clinic.

#### Ketamine and magnesium sulfate

Ketamine will be given according to the protocol described above combined with an infusion of two ampoules of 0.15 g/ml of magnesium sulfate (1.5 g per 10 ml) diluted in 250 ml of physiological saline (0.9% NaCl),

#### Placebo

Physiological saline will be administered in injectable form (0.9% NaCl) in a 250-ml or 45-ml infusion via the electric syringe pump.

#### Regimen

Each patient will receive successively in random order each of the three products with a period of washout between every administration. The duration of the washout will be 5 weeks (35 days). The duration of each infusion is 2½ h per patient as follows:

- *Ketamine-placebo group:* Ketamine will be administered for 2 h and placebo for 30 minutes.
- *Ketamine-magnesium sulfate group:* Ketamine will be administered for 2 h and magnesium sulfate for 30 minutes.
- *Placebo-placebo group:* Placebo will be administered for 2 h and placebo for 30 minutes.

The drugs used in the study (ketamine, ketamine-magnesium, and placebo) are prepared, conditioned, and released in the hospital pharmacy by one qualified person according to good manufacturing principles. Once the treatment is completed, the empty infusion bags are then returned to the packaging box, and the number of infusion bags in each dispensed container is verified and recounted at the end of the treatment by two persons totally independent of the protocol.

### Objective

The primary objective of this study is to compare, using a crossover design, the analgesic efficacy of intravenous ketamine with that of placebo in patients with intractable NP. Secondary objectives are as follows:

- To compare the additive analgesic efficacy of magnesium and ketamine
- To study the time course of pain and analgesia after intravenous administration of ketamine, ketamine-magnesium, and placebo

### Eligibility

#### Inclusion criteria

1. Patients ≥ 18 years old
2. Patients with chronic pain (for > 3 months), having the characteristics of a peripheral or central neuropathy justifying implementation of a therapeutic program with courses of intravenous ketamine injection
3. Patients who have never received ketamine infusion for care of their NP
4. History of illness compatible with an injury or disease of the somatosensory system
5. Localized pain in a neuroanatomical territory
6. Neurological examination shows sensory abnormalities
7. For women of childbearing age, they will be enrolled in the study after a negative urine pregnancy test; in case of suspicion of pregnancy, a blood pregnancy test should be performed.
8. Cooperation and willingness to follow the study
9. Acceptance to give written consent
10. Affiliated with the French social security system
11. Inscription or acceptance of inscription in the national register of volunteers involved in trials

#### Exclusion criteria

1. Patients who have received an intravenous ketamine infusion
2. Patients with one or many contraindications to ketamine administration: known hypersensitivity to ketamine in which one of the constituents of the product, uncontrolled high blood pressure, severe cardiac insufficiency
3. Patients with one or many contraindications to magnesium sulfate administration: patients with severe renal impairment
4. Patients with one or many contraindications to administration of sodium chloride: water inflation, fluid retention
5. Patients with a medical history and/or surgical history judged by the investigator not to be consistent with the clinical trial
6. Patients with drug treatments judged by the investigator not to be consistent with the clinical trial
7. Pregnant or lactating women
8. Patient who participated in another clinical trial, located in the exclusion period, or received benefits > €4500 during 12 months before the beginning of the trial
9. Patients with cooperation and understanding not strictly adherent to the conditions of the clinical trial
10. Patients receiving a measure of legal protection
11. Patients not affiliated with the French social security system

### Definition of outcome measures

#### Outcome measures

The primary outcome is the AUC of pain intensity assessed on a 0–10 NPRS for a period of 5 weeks (35 days) from the day of intravenous treatment.

#### Secondary outcome measure

**Fig. 2:**
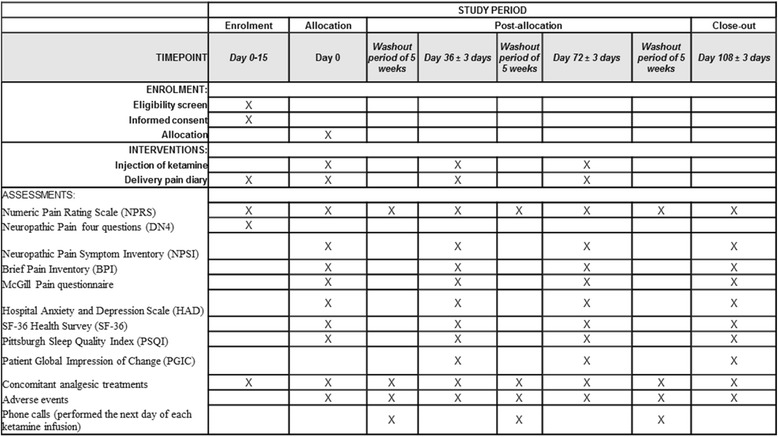
Summary of assessments

Secondary endpoints are the evaluation of pain (NPRS, NPSI, BPI, McGill Pain Questionnaire, PGIC), anxiety and depression (HADS), quality of life (SF-36), and quality of sleep (PSQI) using a variety of scales and questionnaires. Furthermore, the intensity of the average and maximum pain scores as well as concomitant analgesic treatments will be reported in a daily pain diary. The various assessments and questionnaires are summarized in Fig. 2.

### Recruitment

This clinical trial will be carried out by the physicians of the pain clinic and of the clinical pharmacology center/CIC Inserm 1405 of CHU Clermont-Ferrand in charge of the project. The number of subjects needed (*n* = 22) is quite realistic according to the recruitment capacities of the pain clinic. The duration of recruitment is expected to be 2 years.

### Allocation concealment

On the day of the first visit, inclusion and exclusion criteria are verified, and written informed consent is obtained by the physician. A clinical nurse independent from the protocol obtains the randomization number from the hospital pharmacy, and the patient is then randomized into one of the three groups. The assignment to the treatments will follow a predetermined randomization list and is generated using random blocks by a clinical research associate totally independent from the protocol. The randomization list and a copy will be edited, placed in a sealed envelope, and handed over to the Clermont-Ferrand University Hospital Pharmacy and to the coordinating center, the Clinical Research Center of Clermont-Ferrand University Hospital.

### Blinding

In order to maintain blinding, packaging will be identical. The clinical research center and the pain clinic are situated on the same floor of the Clermont-Ferrand University Hospital. A clinical nurse of the clinical research center independent from the protocol prepares and gives the treatment to the patient in a room in the pain clinic. The persons who administer and provide clinical supervision are the medical team affiliated with the pain clinic of the University Hospital of Clermont-Ferrand. The person who performs all questionnaires and analyses is not involved in other aspects of the protocol. Furthermore, only ketamine-naïve patients are included in this trial in order to avoid detection of the placebo or the active substance, taking into account the dysphoric effects (even minor) that may accompany ketamine infusion. If an adverse event occurs (e.g., hospitalization or prolonged hospitalization, incapacity or disability), treatment of the enrolled patients will immediately be suspended. The drug dose will not be changed, and treatment will be continued only upon agreement of the sponsor and the investigators if they conclude that the cause of the adverse event is not related or unlikely to be related to the study products. Unblinding will be allowed only in case of a serious adverse event. Only a doctor involved in the protocol but not in the administration or evaluation of the drug will be dealing with such events. All these steps will be conducted and documented according to the procedures of the University Hospital of Clermont-Ferrand and the current regulatory requirements. If an adverse event persists at the end of the study, the investigator will follow the patient until the event is resolved.

### Missing data

Concerning missing data, if the participant misses one of the three treatment blocks, the patient will be excluded from the study. If missing data occur, a sensitivity analysis will be proposed in order to define the level of attrition and the statistical nature of the missing data, and the last data obtained will be reported.

### Data handling and record keeping

We have not established a data and safety monitoring board for this study. However, in order to improve adherence to the intervention, the case report forms will be monitored by a clinical research associate independent from the protocol and will address any safety or other concerns that may arise. During these visits, the following elements will be reviewed: informed consent; compliance with the study protocol and the procedures defined; and quality of data collected in the case report form, including accuracy, missing data, consistency of data with source documents (e.g., medical records, appointment sheets, laboratory results), and management of the pharmaceutical drugs. Then the monitored case report forms will be retained at the data management center (CIC-Inserm 1405, Clermont-Ferrand, France) in a secure, locked room. Then data will be entered by double data entry by two people independent from the protocol. The blocked database will be transferred to the statistician responsible for the statistical analysis. All original records (consent forms, case report forms, questionnaires, and pain diaries) will be kept at the trial site for 15 years. All data will be anonymized and identified by a participant code.

### Sample size calculation

To highlight the analgesic efficacy of intravenous ketamine in patients with intractable NP, sample size estimation was based on a pilot study done at the pain clinic of the University Hospital of Clermont-Ferrand (administration of intravenous ketamine in a similar open-label population), showing an AUC (28 days) of the intensity of pain of 164 ± 38 (unpublished data). Furthermore, in a study comparing similar treatments (ketamine and placebo) in a different chronic pain setting (CRPS), the AUC graph estimate suggested a 35% reduction in AUC (at 28 days), that 18 patients are needed to detect a minimum difference of 57 in the primary outcome, with a two-sided type I error of 1.67% (to take account of multiple comparisons), statistical power of 90%, and an intraindividual correlation coefficient of 0.5 (owing to the crossover design and no carryover effect assumed) [46]. Considering data from our center, a recent study [46] (NCT01602185) currently submitted to a peer-reviewed journal but not yet published clearly shows that pain evaluation remains the same from 28 days to 60 days. Ultimately, allowing for patient dropout for adverse events or premature withdrawal, we plan to recruit 22 subjects.

### Statistical analysis

Statistical analysis will be performed on an intention-to-treat basis using Stata software (version 13; StataCorp, College Station, TX, USA) for a two-sided type I error at α = 5%. Continuous data will be described as mean ± SD or median (IQR) according to the statistical distribution (assumption of normality studied by Shapiro-Wilk test). Categorical parameters will be described as number and percent. The primary endpoint (AUC of pain intensity based on NPRS) will be compared between groups by repeated measures analysis of variance (ANOVA) for crossover designs, taking account of the following effects: treatment group (ketamine versus placebo), sequence, subject (as random effect), and carryover. Then, the “sequence × treatment” interaction will be tested; if it is significant, statistical analysis will cover only the first period of the crossover study. The normality of residuals will be studied. When quantitative endpoints do not meet the normality assumption, a nonparametric paired Wilcoxon test will be used. Sensitivity analysis will determine the statistical nature of missing data to apply the most appropriate imputation approach.

Statistical analyses of secondary outcomes (NPSI, BPI, HAD, SF-36, PSQI and McGill Pain Questionnaire scores) will be performed similarly to those for the primary endpoint. For categorical parameters (PGIC, analgesic response), the Stuart-Maxwell test for proportion paired data or a generalized linear mixed model will be used, taking the above effects into account. Random-effects models, useful to model between- and within-subject variability, will be performed for the effects described for the crossover ANOVA, to compare the additive analgesic efficacy of associating magnesium sulfate versus placebo to ketamine, and to study the evolution of pain and analgesia with the waning of the intravenous administration. In case of omnibus *p* value < 0.05, a post hoc analysis for multiple comparisons (inflation of type I error) will be applied using the Tukey-Kramer test. For non-crossover comparisons, the usual statistical tests will be performed: (1) for quantitative parameters, ANOVA or Kruskal-Wallis test if the conditions for parametric tests are not met (normality and homoscedasticity on Fisher-Snedecor test); and (2) for categorical variables, the chi-square test or Fisher’s exact test. When appropriate (*p* < 0.05), an appropriate post hoc test will be applied: Tukey-Kramer post hoc ANOVA or Dunn post hoc Kruskal-Wallis. As discussed by Feise [47], type I error (α) will not be adjusted systematically, but rather on a case-by-case basis, in the light of clinical rather than exclusively statistical considerations.

## Discussion

Persistent pain is difficult to treat, and a range of evidence indicates that NMDARs play an important role in sensitization, windup, and neuroplastic changes in the central nervous system [48]. Ketamine may reduce NP symptoms via its antagonistic action on NMDARs [49–51]. Magnesium, a physiological blocker of the NMDAR calcium channels, has also been shown to be effective in the treatment of NP, such as postherpetic neuralgia, CRPS, and phantom limb pain [51–53]. Researchers in two studies examined the effect of these antagonists, and the results were contradictory. Felsby et al. [49] reported that ketamine was more effective than magnesium in the treatment of chronic NP, whereas Kim et al. [51] did not find a significant difference between the two. This discrepancy may have been due to a difference in magnesium dose and administration time. Ketamine and magnesium were given separately but never in combination, and the major limitation of these clinical studies is that they had no placebo group. The objective of the present study is to compare three different forms of injection (ketamine alone, ketamine-magnesium, and placebo) to optimize analgesia in patients with severe NP. Although ketamine, associated with magnesium sulfate or not, is often administered for NP, physicians have no reliable guidelines for prescribing it.

Furthermore, it is also widely reported that NP is known to impact quality of life with psychological discomfort, anxiety, and/or depression [54]. Recently, an open-label study showed that oral administration of ketamine improved both depression and anxiety symptoms [55]; a single injection was sufficient to reduce refractory depressive symptoms after 2 h and for 1 week [56]. The present clinical trial will also evaluate the efficacy of the three injections on the emotional component of pain and on the comorbidities associated with chronic pain.

The benefit expected for participating patients lies in better follow-up of pain and quality of life, with no additional risk. This double-blind randomized controlled clinical trial will provide objective data on the efficacy of ketamine in the treatment of NP and comorbidities associated with chronic pain, such as anxiety and depression. It will enrich good practice recommendations for ketamine, an anesthetic that is often used off-label for NP.

### Trial status

Recruitment started in November 2015 and is currently ongoing.

## Additional file

Additional file 1: SPIRIT 2013 checklist: recommended items to address in a clinical trial protocol and related documents. (DOC 121 kb)

## Abbreviations

ANOVA: Analysis of variance
BPI: Brief Pain Inventory
CRPS: Complex regional pain syndrome
DN4: Diagnosing Neuropathic Pain 4 Questions
HADS: Hospital Anxiety and Depression Scale
NMDAR: *N*-methyl-d-aspartate receptor
NP: Neuropathic pain
NPRS: Numeric Pain Rating Scale
NPSI: Neuropathic Pain Symptom Inventory
PGIC: Patient Global Impression of Change
PSQI: Pittsburgh Sleep Quality Index
SF-36: 36-item Short Form Health Survey
SPIRIT: Standard Protocol Items: Recommendations for Interventional Trials
